# Reduced Risk of Malaria Parasitemia Following Household Screening and Treatment: A Cross-Sectional and Longitudinal Cohort Study

**DOI:** 10.1371/journal.pone.0031396

**Published:** 2012-02-03

**Authors:** Catherine G. Sutcliffe, Tamaki Kobayashi, Harry Hamapumbu, Timothy Shields, Sungano Mharakurwa, Philip E. Thuma, Thomas A. Louis, Gregory Glass, William J. Moss

**Affiliations:** 1 Department of Epidemiology, Johns Hopkins Bloomberg School of Public Health, Baltimore, Maryland, United States of America; 2 W. Harry Feinstone Department of Molecular Microbiology and Immunology, Johns Hopkins Bloomberg School of Public Health, Baltimore, Maryland, United States of America; 3 Department of Biostatistics, Johns Hopkins Bloomberg School of Public Health, Baltimore, Maryland, United States of America; 4 Malaria Research Trust, Choma, Zambia; Menzies School of Health Research, Australia

## Abstract

**Background:**

In regions of declining malaria transmission, new strategies for control are needed to reduce transmission and achieve elimination. Artemisinin-combination therapy (ACT) is active against immature gametocytes and can reduce the risk of transmission. We sought to determine whether household screening and treatment of infected individuals provides protection against infection for household members.

**Methodology/Principal Findings:**

The study was conducted in two areas in Southern Province, Zambia in 2007 and 2008/2009. To determine the impact of proactive case detection, households were randomly selected either to join a longitudinal cohort, in which participants were repeatedly screened throughout the year and those infected treated with artemether-lumefantrine, or a cross-sectional survey, in which participants were visited only once. Cross-sectional surveys were conducted throughout the year. The prevalence of RDT positivity was compared between the longitudinal and cross-sectional households at baseline and during follow-up using multilevel logistic regression. In the 2007 study area, 174 and 156 participants enrolled in the cross-sectional and longitudinal groups, respectively. In the 2008/2009 study area, 917 and 234 participants enrolled in the cross-sectional and longitudinal groups, respectively. In both study areas, participants and households in the longitudinal and cross-sectional groups were similar on demographic characteristics and prevalence of RDT positivity at baseline (2007: OR = 0.97; 95% CI:0.46, 2.03 | 2008/2009: OR = 1.28; 95% CI:0.44, 3.79). After baseline, the prevalence of RDT positivity was significantly lower in longitudinal compared to cross-sectional households in both study areas (2007: OR = 0.44; 95% CI:0.20, 0.96 | 2008/2009: OR = 0.16; 95% CI:0.05, 0.55).

**Conclusions/Significance:**

Proactive case detection, consisting of screening household members with an RDT and treating those positive with ACT, can reduce transmission and provide indirect protection to household members. A targeted test and treat strategy could contribute to the elimination of malaria in regions of low transmission.

## Introduction

An estimated 225 million cases of malaria and 781,000 deaths occurred worldwide in 2009, with the majority in the African region [1]. In the past decade, international support and funding for malaria control increased dramatically and targets were set to increase coverage of interventions to over 80% by 2010, reduce the burden of malaria by 75% by 2015 [1], and eliminate malaria in 8–10 countries by 2015 [1]. This renewed commitment to malaria elimination has been made possible with increased coverage of four key interventions: insecticide-treated nets (ITNs), indoor residual spraying (IRS) of targeted households, treatment with artemisinin-combination therapy (ACT), and intermittent preventive treatment for high-risk groups including pregnant women and infants. Programs that have achieved high coverage with these interventions have shown dramatic decreases in the number of malaria cases, admissions and deaths [1], [2], [3], [4], and 11 African countries have demonstrated large (>50%) and sustained decreases in the burden of malaria [1].

Several case detection strategies have been implemented in regions affected by malaria [5]. Many programs rely on identification of symptomatic individuals at healthcare facilities (passive case detection). However, the prevalence of asymptomatic or minimally symptomatic parasitemia in a population can be as high as 35% [6], [7], [8], [9]. As these individuals do not exhibit symptoms severe enough to seek care, their infections go untreated, thereby serving as parasite reservoirs that can maintain transmission. Consequently, additional strategies are needed, particularly in areas of declining transmission. An alternate strategy is to screen individuals for parasitemia and provide treatment to those who are infected (proactive case detection or focal screening and treatment) [10], thereby identifying and treating asymptomatic or minimally symptomatic individuals. Use of ACT can enhance this strategy as it is active against immature gametocytes [11]. Treatment not only reduces the burden of parasites within the individual but can reduce the risk of transmission to mosquitoes [12], [13]. In regions of declining transmission, the burden of malaria could potentially be reduced to such an extent that elimination is achievable.

Using a series of longitudinal and cross-sectional household surveys in a setting of declining malaria transmission in southern Zambia, we sought to quantify the effect of proactive case detection by screening individuals within households and treating those infected with ACT. We hypothesized that the prevalence of infection would be lower in households in the longitudinal cohort where individuals were repeatedly tested and treated compared to households in the cross-sectional surveys where individuals were tested and treated once.

## Methods

The study was approved the University of Zambia Research Ethics Committee and the Institutional Review Board at the Johns Hopkins Bloomberg School of Public Health.

### Study design

This study was conducted within the context of an epidemiologic study to determine changes in the prevalence of parasitemia and gametocytemia in a region of unstable malaria transmission in southern Zambia [14], [15]. To determine the impact of proactive case detection, households were randomly selected either to join a longitudinal cohort, in which participants were repeatedly screened throughout the year and those infected treated with ACT, or a cross-sectional survey, in which participants were visited only once. A series of cross-sectional surveys was conducted throughout the year to account for any temporal and seasonal changes in transmission.

### Study site

The study was conducted in the catchment area of Macha Hospital in Choma District, Southern Province, Zambia between April 2007 and December 2009. Macha Hospital is located approximately 70 km from the nearest town of Choma on a plateau at an altitude of approximately 1,100 meters above sea level and in a habitat characterized as Miombo woodland. There is a single rainy season from approximately November through April, followed by a cool, dry season from April to August, and a hot, dry season from August to November. The catchment area is populated by traditional villagers living in small, scattered homesteads. *Anopheles arabiensis* is the primary vector responsible for malaria transmission [16], which peaks during the rainy season.

The Southern Province of Zambia historically had hyperendemic transmission of *Plasmodium falciparum* [17]. More recently, the entomological inoculation rate for *An. arabiensis* was estimated to range from 1.6 to 18.3 infective bites per person per season [16] and the number of children hospitalized for malaria decreased dramatically (unpublished data). Zambia introduced artemether-lumefantrine as antimalarial therapy in 2002, and insecticide-treated bed nets (ITNs) were widely distributed in Southern Province, Zambia in 2007 [14]. Widespread IRS has not been formally conducted in the study area.

**Figure 1:**
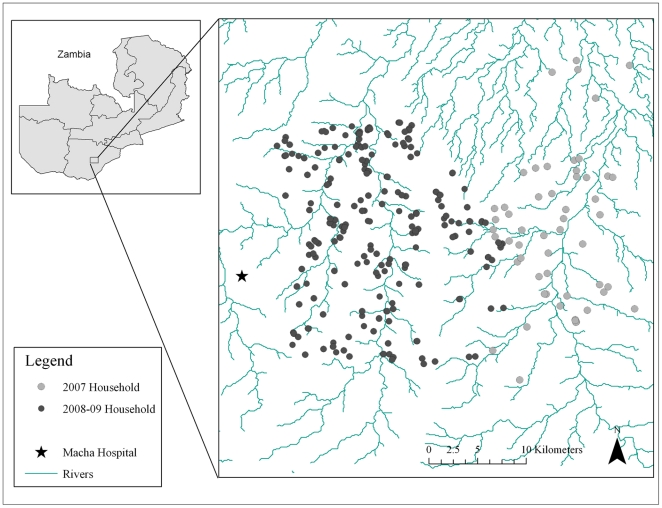
Map of the study sites in Choma district, Southern province, Zambia.

The study site in 2007 consisted of a 525 km^2^ region east of Macha Hospital (Figure 1). In 2008 and 2009, the study site was shifted to an adjacent 575 km^2^ area west of the 2007 study site and closer to Macha Hospital. This was done for logistical reasons as distances to the study households proved to be difficult to navigate operationally, particularly during the rainy season when roads and bridges were flooded.

### Randomization

At the beginning of the study in each site, satellite images were used to construct a sampling frame for the random selection of households to be enrolled in a prospective longitudinal cohort study and serial cross-sectional surveys. The sampling frame was constructed from a Quickbird™ satellite image obtained from DigitalGlobe Services, Inc. (Denver, Colorado). The image was imported into ArcGIS 9.2 (Redlands, CA) and locations of households were identified and enumerated manually. In this area, households consist of one or more domestic structures where members of a family or extended family reside. Operationally, structures of appropriate size and shape situated within cleared sections of land approximately 50 meters wide were identified as potential residences from the satellite image. Satellite images have been used successfully to establish a sampling frame in Zambia [18]. The desired number of households was randomly selected from the sampling frame to be enrolled in the longitudinal cohort study. From the remaining households, the desired number of households was randomly selected without replacement to be enrolled in each of the cross-sectional surveys conducted throughout the calendar year. Random selection was performed to ensure as much as possible that longitudinal and cross-sectional households were comparable at baseline.

### Study procedures

Study procedures were the same for households enrolled in the longitudinal cohort and cross-sectional surveys and began with community mobilization activities, including approvals from local chiefs and headmen. A field team was provided with images and coordinates of the randomly selected households. All individuals within a household were enumerated and were eligible to participate. Permission was obtained from the head of household and written informed consent was obtained from each individual present in the household. The consent process included a description of their participation in either the longitudinal cohort or cross-sectional survey. Not all participants in a household were required to participate.

During each study visit, a questionnaire was administered to each consenting participant older than 18 years of age residing within the household and to parents or guardians of those younger than 18 years of age. Data collected included demographic information, history of recent malaria and antimalarial treatment, knowledge of malaria transmission and prevention, and the use of ITNs. Participant's temperature was measured using a Braun Thermoscan® ear thermometer. A blood sample was collected by finger prick from consenting participants for a rapid diagnostic test (RDT) for malaria. The RDT (ICT Diagnostics, Cape Town, South Africa) detected *P. falciparum* histidine-rich protein 2 and was shown to detect 82% of test samples with wild-type *P. falciparum* at a concentration of 200 parasites/µL and 98% of test samples with a concentration of 2000 parasites/µL, with false positives in 0.6% of negative samples [19]. Individuals who were RDT positive were offered treatment with artemether-lumefantrine (Coartem®) according to national guidelines.

**Figure 2:**
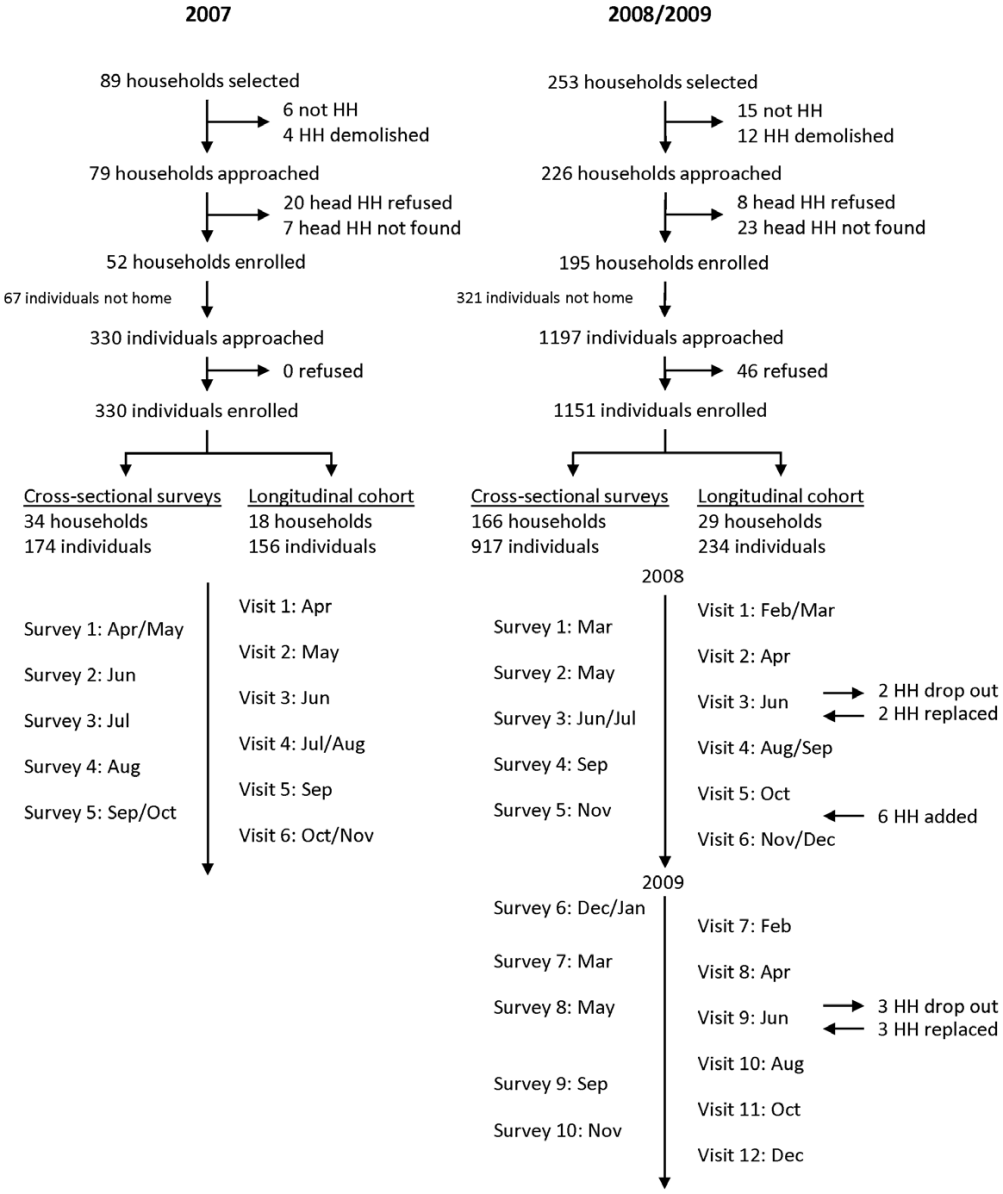
Study flow diagram for 2007 and 2008/2009. HH: household.

Households selected for the longitudinal cohort were repeatedly surveyed approximately five times per calendar year (Figure 2). In the event that households withdrew or moved, replacement households were randomly selected from the sampling frame. In 2007 and 2008, the target sample size for the cohort was approximately 17 households. At the end of 2008, additional households were selected to increase the sample size to 24 in 2009.

Households selected for each cross-sectional survey were visited only once. Cross-sectional surveys were carried out approximately five times per calendar year. The target sample size for each cross sectional survey was the same as the target sample size per month of the corresponding cohort. For logistical reasons, the longitudinal and cross-sectional surveys were conducted in alternating months beginning April 2007 and February 2008.

### Statistical methods

The primary endpoint was the point prevalence of RDT positivity in the household. Analyses were conducted at two time points: 1) at baseline to determine whether the prevalence of RDT positivity differed between longitudinal and cross-sectional households at the first study visit prior to antimalarial treatment; and 2) during follow-up to determine whether the prevalence of RDT positivity differed between the longitudinal households throughout follow-up (with continued exposure to treatment of RDT positive individuals) and the cross-sectional households. For the baseline analysis, all study visits for participants in the cross-sectional households but only the first study visit for participants in the longitudinal households were included. For the follow-up analysis, all study visits for participants in the cross-sectional households were included but, for participants in the longitudinal households, the first study visit was excluded and only subsequent study visits were included.

For the primary analysis at both baseline and follow-up, logistic regression with a random intercept for households and robust standard error estimation was used to estimate the odds ratio of RDT positivity for the longitudinal compared to the cross-sectional households as randomized. Within-participant clustering was considered during follow-up, as participants in longitudinal households contributed multiple visits. However, the within-participant, intraclass correlation coefficient (ICC) was low (ICC = 0.00002); therefore, only within-household clustering is reported. Transmission season, high or low, was included in the models to account for temporal changes, and was defined based on rainfall data and pediatric hospitalizations for malaria at Macha Hospital during the study periods. Within a calendar year, the high transmission season was assumed to occur from January 1 to June 30 and from November 1 to December 31. For the 2008/2009 study period, season was defined across calendar years, such that high transmission seasons occurred from January 1 to June 30, 2008, November 1, 2008 to June 30, 2009, and November 1 to December 31, 2009. As RDT positivity was relatively common in 2007, the analysis was repeated using Poisson regression with robust standard error estimation (Table S1), which yielded similar inferences.

As a secondary analysis at both baseline and follow-up, multilevel logistic regression with a random intercept for households and robust standard error estimation was used to explore the contribution of individual and household level baseline characteristics. A two-level model was fit with individuals (level 1) nested within households (level 2). Both individual-level (e.g. age, sex, ITN use) and household-level (e.g. housing type, season) covariates that differed (p<0.10) between groups at baseline were considered for inclusion in the models.

## Results

### Characteristics of the study population at the first study visit

In the 2007 study area, 174 participants from 34 households were enrolled in the cross-sectional group and 156 participants from 18 households were enrolled in the longitudinal group (Table 1 and 2; Figure 2). The characteristics of participants in each group were similar in terms of sex (% male: longitudinal[L]:42.3%; cross-sectional[C]:50.0%; p = 0.16), age (% <5 years: L:25.6%; C:21.1%; p = 0.30), and education (% adults with some secondary education: L:81.4%; C:70.4%; p = 0.35). The majority of participants did not own an ITN (L:61.5%; C:56.9%; p = 0.36). Participants differed significantly on their reporting of antimalarial use in the prior 2 weeks (L:12.2%; C:4.6%; p = 0.03), recent visit to a health center or post for an illness (L:50.0% less than one month ago; C:31.0%; p = 0.01) and for their last febrile episode (L:57.1%; C:30.5%; p<0.0001), and sleeping under an ITN at the time of the visit, among participants who owned an ITN (L:70.0%; C:25.3%; p<0.0001).

**Table 1:** Participant characteristics at the initial study visit by year.

	2007			2008&2009		
	Cross-sectional N (%)	Longitudinal N (%)	p-value^b^	Cross-sectional N (%)	Longitudinal N (%)	p-value^b^
Number of participants	174	156		917	234	
Male sex	87 (50.0)	66 (42.3)	0.16	438 (47.8)	111 (47.4)	0.93
Median age	13.1 (5.6, 34.7)	12.7 (4.9, 27.6)	0.26	14.3 (6.6, 34.1)	13.0 (5.6, 24.2)	0.13
0–4	36 (21.1)	40 (25.6)		176 (19.4)	50 (21.6)	
5–17	67 (39.2)	57 (36.5)		356 (39.2)	95 (41.0)	
≥18	71 (40.8)	59 (37.8)	0.56	383 (41.9)	89 (38.0)	0.54
Education (among participants ≥18 years)						
< Grade 1	2 (2.8)	1 (1.7)		15 (3.8)	5 (5.8)	
Grade 1–6	19 (26.8)	10 (17.0)		122 (31.1)	24 (27.6)	
Grade 7–12, or higher	50 (70.4)	48 (81.4)	0.35	255 (65.1)	58 (66.7)	0.62
Number of ITN in the household						
0	99 (56.9)	96 (61.5)		311 (33.9)	49 (20.9)	
1	48 (27.6)	44 (28.2)		542 (59.1)	171 (73.1)	
≥2	27 (15.5)	16 (10.3)	0.36	64 (7.0)	14 (6.0)	0.0003
Sleeps under ITN, among participants with ITN	19 (25.3)	42 (70.0)	<0.0001	335 (55.3)	117 (63.2)	0.06
ITN ever treated, among those sleeping under ITN	2 (10.5)	11 (26.2)	0.21	63 (18.8)	34 (29.1)	0.10
House has ever been sprayed with insecticide	3 (1.7)	3 (1.9)	0.63	14 (1.5)	6 (2.6)	0.28
Used any antimalarial medication in last 2 wks	8 (4.6)	19 (12.2)	0.03	29 (3.2)	5 (2.1)	0.41
Presence of any symptoms in last 48 hours^a^	128 (73.6)	93 (59.6)	0.01	543 (59.2)	135 (57.7)	0.67
Self-reported fever in the last 48 hrs	72 (41.4)	51 (32.7)	0.10	218 (23.8)	61 (26.1)	0.46
Fever (≥38°C)	7 (4.1)	7 (4.5)	0.83	16 (1.7)	2 (0.9)	0.33
Visited health post/center/hospital for last fever	53 (30.5)	89 (57.1)	<0.0001	586 (63.9)	126 (53.9)	0.005
Last visited a health center or post for an illness						
< = 1 month ago	54 (31.0)	78 (50.0)		336 (36.6)	71 (30.3)	
2–6 months ago	52 (29.9)	34 (21.8)		275 (30.0)	86 (36.8)	
>6 months ago	46 (26.4)	32 (20.5)		171 (18.7)	41 (17.5)	
Do not know	22 (12.6)	12 (7.7)	0.01	135 (14.7)	36 (15.4)	0.17
RDT positive	41 (23.6)	38 (24.4)	0.87	36 (3.9)	10 (4.3)	0.81

ITN: insecticide-treated net.

a Symptoms included fever, chills, headache, diarrhea, cough, nausea/vomiting.

b Comparison of cross-sectional and longitudinal participants using the chi-square test for binary characteristics and the Wilcoxon ranksum test for continuous characteristics.

**Table 2:** Household characteristics at the initial study visit by year.

	2007			2008&2009		
	Cross-sectional N (%)	Longitudinal N (%)	p-value^a^	Cross-sectional N (%)	Longitudinal N (%)	p-value^a^
Number of households	34	18		166	29	
Median number of participants per household (range)	5 (1–11)	8 (3–19)	0.006	5 (1–21)	8 (2–15)	<0.0001
Source of water						
Private well or pump	2 (5.9)	3 (16.7)		2 (1.2)	1 (3.5)	
Public well, pump or standpipe	19 (55.9)	11 (61.1)		100 (60.2)	15 (51.7)	
River or stream	12 (35.3)	2 (11.1)		37 (22.3)	6 (20.7)	
Unprotected well	1 (2.9)	2 (11.1)	0.14	27 (16.3)	7 (24.1)	0.57
Toilet						
Pit latrine	16 (47.1)	8 (44.4)		120 (72.3)	15 (51.7)	
No facility/bush/field	18 (52.9)	9 (50.0)		46 (27.7)	14 (48.3)	
Other	0 (0.0)	1 (5.6)	0.38	0 (0.0)	0 (0.0)	0.03
Source of light						
Candle	4 (11.8)	0 (0.0)		27 (16.3)	0 (0.0)	
Lantern	28 (82.4)	17 (94.4)		117 (70.5)	27 (93.1)	
Other	2 (5.9)	1 (5.6)	0.31	22 (13.3)	2 (6.9)	0.03
Material of floor						
Cement	13 (38.2)	5 (27.8)		35 (21.1)	5 (17.2)	
Earth	21 (61.8)	13 (72.2)	0.45	131 (78.9)	24 (82.8)	0.64
Material of walls						
Fired brick/cement	28 (82.4)	16 (88.9)		148 (89.2)	27 (93.1)	
Other	6 (17.7)	2 (11.1)	0.53	18 (10.8)	2 (6.9)	0.52
Material of roof						
Iron sheets/corrugated tin	9 (26.5)	3 (16.7)		44 (26.5)	8 (27.6)	
Pole and grass	23 (67.7)	13 (72.2)		119 (71.7)	20 (69.0)	
Other	2 (5.9)	2 (11.1)	0.62	3 (1.8)	1 (3.5)	0.84

a Comparison of cross-sectional and longitudinal households using the chi-square test for binary characteristics and the Wilcoxon ranksum test for continuous characteristics.

In the 2008/2009 study area, 917 participants from 166 households were enrolled in the cross-sectional group and 234 participants from 29 households were enrolled in the longitudinal group (Table 1 and 2; Figure 2). The characteristics of participants in each group were also similar in terms of sex (% male: L:47.4%; C:47.8%; p = 0.92), age (% <5 years: L:21.6%; C:19.4%; p = 0.52), and education (% adults with some secondary education: L:66.7%; C:65.1%; p = 0.62). Longitudinal and cross-sectional participants differed in their ownership (L:79.1% with ≥1 ITN; C:66.1%; p = 0.0003) and use of ITNs (L:63.2%; C:55.3%; p = 0.06), and their use of health services for their last febrile episode (L:53.9%; C:63.9%; p = 0.005).

### Comparison of RDT positivity between cross-sectional and longitudinal groups at the first study visit prior to treatment exposure

**Figure 3:**
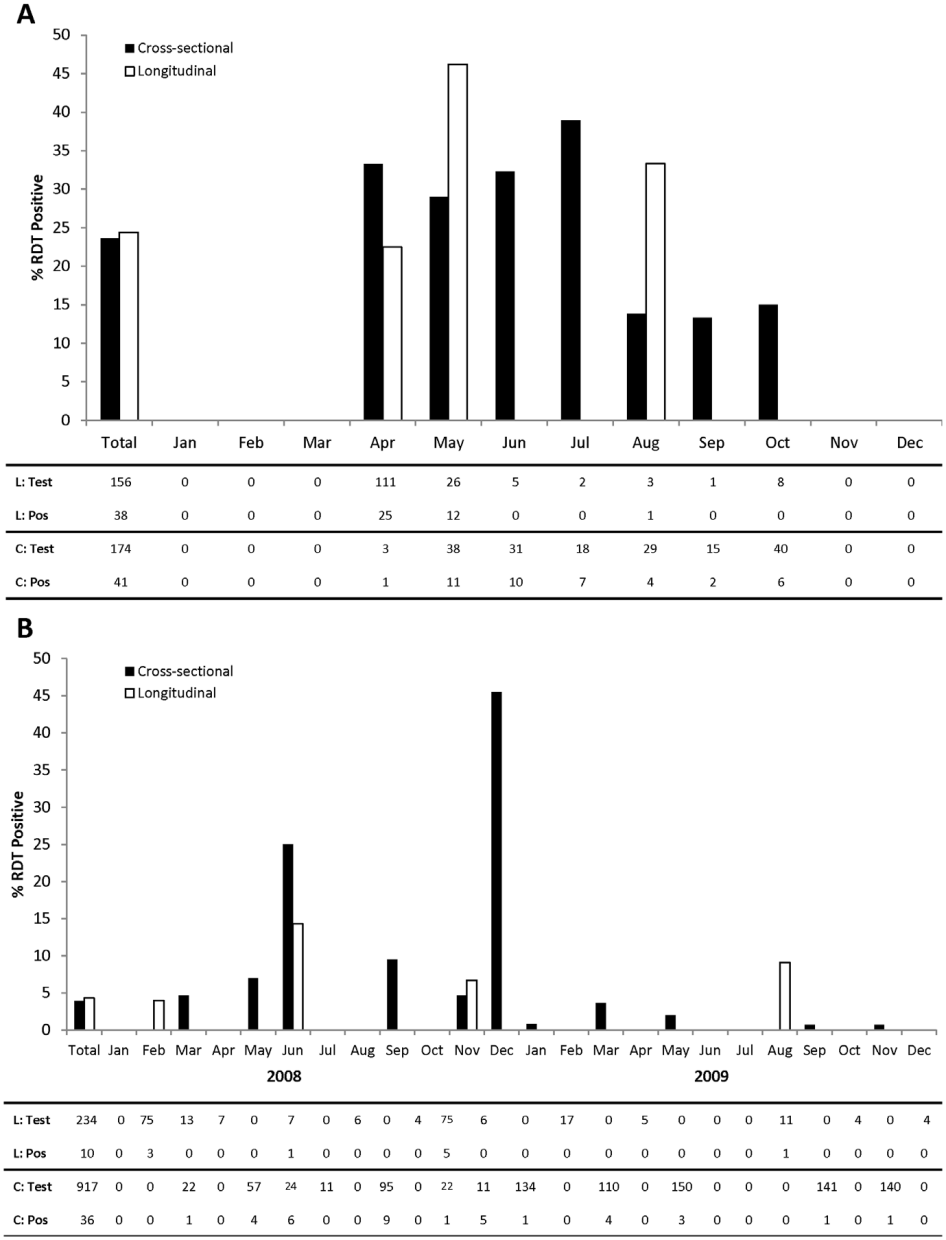
Percent RDT positive at the initial study visit by group and month in (A) 2007 and (B) 2008/2009.

In the 2007 study area, 41 participants (23.6%) from 21 households (61.8%) were RDT positive in the cross-sectional group, and 38 participants (24.4%) from 14 households (77.8%) were RDT positive in the longitudinal group (Figure 3A). Among cross-sectional households with at least one RDT positive individual, the median number of positive individuals was 2 (IQR:1, 3) for a median household prevalence of 30.0% (IQR:25.0, 50.0). Among longitudinal households with at least one RDT positive individual, the median number of positive individuals was 1.5 (IQR:1, 3) for a median household prevalence of 33.3% (IQR:12.5, 44.4). In both groups, the majority of infections were in children younger than 15 years. Only 10% of individuals who were RDT positive had a documented fever at the study visit, with the majority of symptomatic infections occurring among children 5–17 years of age.

In the primary analysis, no differences in the odds of RDT positivity were observed between households in the longitudinal cohort and cross-sectional surveys (odds ratio [OR]:0.97; 95% CI:0.46, 2.03) (Table 3). Some clustering of RDT positivity was observed within households (ICC:0.17; σ:0.81). Adjusting for season decreased the OR to 0.59 (95% CI:0.25, 1.39; ICC:0.17; σ:0.83), although this result was not statistically significant.

**Table 3:** Comparison of RDT positivity between longitudinal and cross-sectional households at the initial study visit and during follow-up, by study year.

	2007		2008 and 2009	
Primary Analysis	OR (95% CI)	ICC	OR (95% CI)	ICC
***Comparison at the initial study visit*** a				
Crude model	0.97 (0.46, 2.03)	0.17	1.28 (0.44, 3.79)	0.40
Adjusted for season^b^	0.59 (0.25, 1.39)	0.17	0.90 (0.31, 2.65)	0.35
***Comparison during follow-up*** a				
Crude model	0.44 (0.20, 0.96)	0.18	0.16 (0.05, 0.55)	0.39
Adjusted for season^b^	0.37 (0.16, 0.88)	0.22	0.13 (0.04, 0.41)	0.30
**Secondary Analysis**				
***Comparison at the initial study visit*** a				
Adjusted for season and other baseline individual and household characteristics^c^	0.57 (0.21, 1.53)	0.25	0.85 (0.28, 2.54)	0.34
***Comparison during follow-up*** a				
Adjusted for season and other baseline individual and household characteristics^c^	0.32 (0.13, 0.80)	0.25	0.12 (0.04, 0.39)	0.31

a Comparison at initial study visit included all participants in the cross-sectional group and the first study visit for participants in the longitudinal group; comparison during follow-up included all participants in the cross-sectional group and excluded the first study visit for participants in the longitudinal group.

b For 2007, the transmission season was defined as Jan-Jun and Jul-Oct; for 2008/2009, the transmission season was defined as Jan-Jun 2008, Jul-Oct 2008, Nov 2008-Jun 2009, Jul-Oct 2009, and Nov-Dec 2009.

c For 2007, other baseline characteristics included age, sleeping under an ITN and taking an antimalarial in the last 2 weeks; for 2008/2009, other baseline characteristics included age, sleeping under an ITN, type of toilet and source of light.

In the 2008/2009 study area, 36 participants (3.9%) from 23 households (13.9%) were RDT positive in the cross-sectional group, and 10 participants (4.3%) participants from 7 households (24.1%) were RDT positive in the longitudinal group (Figure 3B). Among cross-sectional households with at least one RDT positive individual, the median number of positive individuals was 1 (IQR:1, 1) for a median household prevalence of 20.0% (IQR:14.3, 33.3). Among longitudinal households with at least one RDT positive individual, the median number of positive individuals was 1 (IQR:1, 2) for a median household prevalence of 12.5% (IQR:11.1, 18.2). In both groups, the majority of infections were in children younger than 15 years. Only 15% of individuals who were RDT positive had a documented fever at the study visit, with the majority of symptomatic infections occurring among children 5–17 years of age.

In the primary analysis, no differences in the odds of RDT positivity were observed between households in the longitudinal cohort and cross-sectional surveys (OR:1.28; 95% CI:0.44, 3.79; ICC:0.40; σ:1.47) (Table 3). Similar to the findings in 2007, adjusting for season decreased the OR to 0.90 (95% CI:0.31, 2.65; ICC:0.35; σ:1.32), although this result was not statistically significant.

In both study areas, accounting for individual and household level characteristics at baseline did not significantly impact the results (Table 3).

### Comparison of RDT positivity between cross-sectional and longitudinal groups during follow-up

In the 2007 study area, 174 participants from 34 households in the cross-sectional group and 126 participants from 17 households in the longitudinal group after their first visit were included in the analysis. Longitudinal participants contributed a median of 2 study visits (range:1, 3) and households were surveyed a median of 3 times (range:1, 4). In the longitudinal group, 38 infections were detected during follow-up from 33 individuals (three with 2 RDT positive results and one with 3 RDT positive results) in 10 households. The participants with multiple positive RDTs were all 5 years of age or younger. Individuals with positive RDTs within the same household were primarily found on the same study visit.

**Figure 4:**
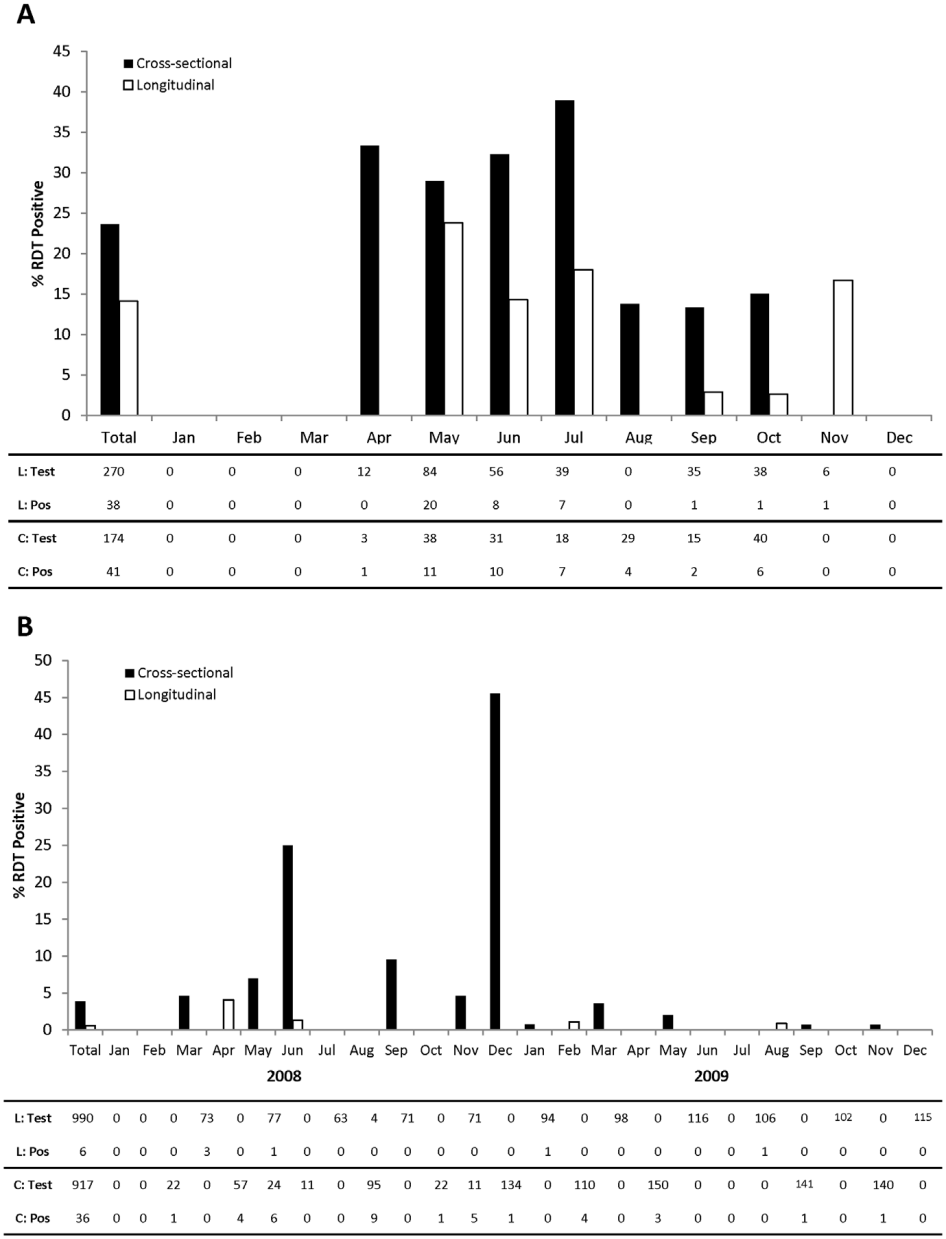
Percent RDT positive during follow-up by study group and month in (A) 2007* and (B) 2008/2009*. *includes all participants in the cross-sectional group and excludes the first study visit for participants in the longitudinal group.

In the primary analysis, the odds of RDT positivity were significantly lower for households in the longitudinal group after the first visit compared to the cross-sectional households (OR:0.44; 95% CI:0.20, 0.96; ICC:0.18; σ:0.86) (Table 3; Figure 4A) and the results remained significant after adjusting for season (OR:0.37; 95% CI:0.16, 0.88; ICC:0.22; σ:0.98).

In the 2008/2009 study area, 917 participants from 166 households in the cross-sectional group and 190 participants from 29 households in the longitudinal group after their first visit were included in the analysis. Longitudinal participants contributed a median of 3 study visits (range:1, 11) and households were surveyed a median of 10 times (range:1, 11). In the longitudinal group, six infections were detected during follow-up from six individuals in five households. The two infections in the same household were detected on different visits.

In the primary analysis, the odds of RDT positivity were significantly lower for households in the longitudinal group after the first visit compared to the cross-sectional households (OR:0.16; 95% CI:0.05, 0.55; ICC:0.39; σ:1.44) (Table 3; Figure 4B). The results remained significant after adjusting for season (OR:0.13; 95% CI:0.04, 0.41; ICC:0.30; σ:1.19). There was some evidence of a lowering of the household malaria risk over time as the odds ratio for RDT positivity was lower in subsequent follow-up visits in the longitudinal households compared to the cross-sectional households, suggesting a cumulative effect (first visit: crude OR = 0.44; 95% CI = 0.10, 1.93; second visit: OR = 0.15; 95% CI = 0.02, 1.45; third visit: OR = 0.16; 95% CI = 0.02, 1.61).

In both study areas, accounting for individual and household level characteristics at baseline did not significantly impact the results (Table 3).

## Discussion

We sought to quantify the effect of a proactive strategy of household screening and treatment with ACT of individuals infected with *Plasmodium falciparum* on the reduction in malaria transmission within households. The prevalence of parasitemia was significantly reduced in households repeatedly screened and treated compared to a control group of households. This effect was consistent across two geographic areas with different levels of malaria transmission. Strategies to reduce and interrupt malaria transmission through treatment of symptomatic and asymptomatic infections are possible with the use of ACT. Artemisinin derivatives are active against both young and mature asexual parasites and immature gametocytes [11], thus preventing gametocyte development and blocking transmission to mosquitoes. Studies have shown that gametocyte carriage is significantly reduced among individuals treated with artemisinin derivatives in comparison to other antimalarials [11], [13], [20], [21], [22]. Artemisinin-based derivatives also have been shown to decrease both transmission to mosquitoes and the prevalence of high density oocyst infections within mosquitoes [12], [13].

In areas where malaria transmission has declined following implementation of effective control measures, additional strategies are needed to identify and treat asymptomatic or minimally symptomatic cases to eliminate reservoirs of infection, interrupt transmission and achieve elimination [5]. Several case detection strategies have been developed and implemented. Passive case detection, involving identification of symptomatic patients seeking care at health facilities, requires the least resources. This strategy, however, does not detect asymptomatic or minimally symptomatic infections as these individuals will not present to health care facilities. The proportion of malaria cases that are asymptomatic or minimally symptomatic is substantial and can be as high as 96% [6], [23], [24], suggesting that the majority of infectious cases would be missed with passive case detection. Reactive case detection [5] extends this strategy based on the observation that malaria cases are spatially clustered and that cases identified at health centers (index cases) represent foci of infection within households and surrounding neighborhoods. With reactive case detection, residents of households of index cases and possibly of households in the immediate vicinity are screened and treated if found to be infected. However, little data exist on the appropriate radius from the index household that should be screened, and this radius likely varies in different epidemiological settings. In a study of reactive case detection in rural southern Zambia, the prevalence of malaria was found to be significantly higher among residents of households of index cases than among residents of randomly selected households in the area [25]. However, even with reactive case detection, many infectious cases would be missed. In our community-based study, only 10–15% of RDT positive individuals were symptomatic with a documented fever and up to 70% of individuals reported they did not seek care for their last febrile episode. Consequently, most infected participants would have been missed through reactive case detection. In 2007, 79 individuals from 35 households were RDT positive at the first study visit; however, only 16 residents of 10 households reported fever and sought care. If these cases sought care and their households were screened, as in reactive case detection, 38 RDT positive individuals would have been detected, representing only 52% of all RDT positive individuals. In 2008/2009, the proportion of RDT positive individuals who would have gone undetected increased to 72%.

A second strategy to target asymptomatic or minimally symptomatic individuals is mass drug distribution, in which case detection is not attempted and drugs are distributed to a population regardless of symptoms and without diagnosis of infection. Mass drug distribution has a long history in malaria control, achieving transient reductions in incidence or prevalence but with little effect on transmission [26].

Lastly, a case detection strategy more intensive than passive or reactive case detection, but without treating the entire population, is proactive case detection [5], where populations are screened and infected individuals are treated (“test and treat”) as was done in this study. This strategy requires substantial resources for personnel and drugs, a rapid diagnostic test with a high positive predictive value, and a non-mobile population willing to accept screening and treatment of minimally symptomatic individuals. Proactive case detection may be an essential strategy to achieve elimination in regions of sub-Saharan Africa where the burden of malaria has been substantially reduced with current control efforts. Proactive case detection has successfully been implemented in Morocco [27], Brazil [28], Taiwan [29], and Southern China [30], where levels of malaria transmission are lower than in much of sub-Saharan Africa. However, these studies evaluated the success of proactive case detection based on the burden of malaria before and after implementation of a case detection program, without concurrent control groups.

To our knowledge, this is the first formal quantification of the effect size of proactive case detection with ACT using a concurrent comparison group. Although the findings were consistent in two adjacent areas with different levels of malaria transmission, the reduction in malaria prevalence appeared to be greater in the study area with a lower level of transmission intensity. Proactive case detection resulted in a six-fold reduction in prevalence in 2008/2009, where the initial parasite prevalence was 4%, but in only a two-fold reduction in 2007, where the initial prevalence was 24%, suggesting that proactive case detection may have greater impact on malaria transmission in areas where current control measures have succeeded in reducing transmission. These results are consistent with the predicted impact of artemisinin-based therapies in different transmission settings [31].

This study had several limitations. First, as a trial could not be conducted for ethical reasons, this observational study was designed as a cohort study with a series of cross-sectional studies for comparison. Households enrolled in the longitudinal cohort study were repeatedly surveyed and it is possible that the behaviors of household residents were influenced by participation in the study. Indeed, ITN use during follow-up was higher for individuals in the cohort compared to the cross-sectional surveys, particularly during the low transmission season (data not shown). Consequently, it is possible that the effect of proactive case detection was overestimated in longitudinal households. In addition, despite the random selection of households, differences between the cross-sectional and longitudinal households and their residents were identified at baseline that may have impacted their risk of malaria, including ownership and use of ITNs. These differences in ITN use were primarily attributable to the seasons in which the longitudinal (high transmission) and cross-sectional (high and low transmission) households were enrolled and were not apparent after adjustment (data not shown). However, the decreased odds of RDT positivity in the longitudinal households at baseline after adjusting for season may indicate that the effect of proactive case detection during follow-up was overestimated. Second, for ethical reasons all RDT positive individuals in both the cross-sectional and longitudinal groups were treated with ACT. Given the potential for ACT to reduce malaria transmission, there may have been an indirect protective effect conferred to residents living in proximity to households where participants were treated. Consequently, the impact of this strategy may have been underestimated. Lastly, the sensitivity of the RDT decreases at lower parasite densities [19], and infections in individuals with low levels of parasitemia could have been missed. We do not expect this misclassification to have occurred differentially between participants in longitudinal and cross-sectional households, and therefore the effect of this strategy may have been underestimated.

In summary, proactive case detection with treatment using ACT can reduce transmission and provide indirect protection to household members. If resources permit, this strategy could be targeted to focal areas of transmission within regions in the pre-elimination phase of malaria control to achieve further reductions in malaria transmission.

## Supporting Information

Table S1 **Comparison of RDT positivity between longitudinal and cross-sectional households at the initial study visit and during follow-up, estimated using logistic and Poisson regression.**
(DOCX)
