# Supplementary material for: Reduced Risk of Malaria Parasitemia Following Household Screening and Treatment: A Cross-Sectional and Longitudinal Cohort Study
Source: PLoS One. 2012 Feb 3;7(2):e31396. doi: 10.1371/journal.pone.0031396 (PMC3272029; doi:10.1371/journal.pone.0031396)
Supplement: Table S1 — Comparison of RDT positivity between longitudinal and cross-sectional households at the initial study visit and during follow-up, estimated using logistic and Poisson regression. (DOCX) [file pone.0031396.s001.docx]

**Table S1. Comparison of RDT positivity between longitudinal and cross-sectional households at the initial study visit and during follow-up, estimated using logistic and Poisson regression**

|  | **Logistic regression^a^**  **OR (95% CI)** | **Poisson regression^a^**  **RR (95% CI)** |
| --- | --- | --- |
| **2007** |  |  |
| ***At the initial study visit*** |  |  |
| Crude model | 0.97 (0.46, 2.03) | 0.99 (0.58, 1.70) |
| Model adjusted for season | 0.59 (0.25, 1.39) | 0.74 (0.44, 1.23) |
| ***During follow-up*** |  |  |
| Crude model | 0.44 (0.20, 0.96) | 0.56 (0.31, 1.01) |
| Model adjusted for season | 0.37 (0.16, 0.88) | 0.51 (0.27, 0.94) |
|  |  |  |
| **2008/2009** |  |  |
| ***At the initial study visit*** |  |  |
| Crude model | 1.28 (0.44, 3.79) | 1.24 (0.51, 3.02) |
| Model adjusted for season | 0.90 (0.31, 2.65) | 0.91 (0.32, 2.64) |
| ***During follow-up*** |  |  |
| Crude model | 0.16 (0.05, 0.55) | 0.18 (0.07, 0.46) |
| Model adjusted for season | 0.13 (0.04, 0.41) | 0.15 (0.06, 0.36) |

^a^ Logistic and Poisson regression models included a random intercept for households and robust standard error estimation
